# The Enigmatic Mass Over the Clavicle: A Case Report Unveiling a Rapidly Growing Spindle Cell Lipoma

**DOI:** 10.7759/cureus.92718

**Published:** 2025-09-19

**Authors:** Ron Jako Domingo, Daniel Hahn, Andrew Hauptman, Benjamin Kashan, Vinay Tak

**Affiliations:** 1 Surgery, Touro College of Osteopathic Medicine, New York, USA; 2 Surgery, St. Mary's General Hospital, Passaic, USA; 3 Cardiothoracic Surgery, St. Mary's General Hospital, Passaic, USA

**Keywords:** benign mass, lipoma, spindle cell lipoma, supraclavicular mass, surgical excision

## Abstract

Spindle cell lipoma (SCL) is a rare, benign, adipocytic tumor typically located in the subcutaneous tissues of the neck, shoulder, and upper back regions. A 64-year-old female patient presented with a small, soft mass over her left clavicle. Her medical history included well-controlled hypertension and hyperlipidemia, and she had previously undergone an appendectomy and excision of a soft tissue cyst. On follow-up approximately one year later, the mass had demonstrated significant and rapid growth. Surgical excision of the mass revealed a 7.0x6.0x5.0 cm yellow-colored tumor, confirmed as an SCL on histopathological examination. Although benign, this case highlights the potential for rapid growth in SCLs, warranting their inclusion in the differential diagnosis when evaluating masses in the shoulder and neck region.

## Introduction

Spindle cell lipoma (SCL) is a rare, benign adipocytic neoplasm primarily found in the subcutaneous tissues of the posterior neck, upper back, and shoulder regions. These tumors typically manifest as small (<5.0 cm), mobile, slow-growing, and painless masses [1]. Epidemiologically, SCLs show a strong male predominance, with peak incidence occurring between the fifth and seventh decades of life [1,2]. 

Histopathologically, SCLs are distinguished by the presence of florette-like multinucleated giant cells intermixed with coarse eosinophilic collagen bundles [3]. Immunohistochemical analysis of SCLs typically reveals strong positivity for CD34, a marker of hematopoietic progenitor cells, and vimentin, a class III intermediate filament protein commonly expressed in mesenchymal cells [4,5]. Other resources have utilized MRI to aid diagnostic differentiation, demonstrating a mixture of adipose tissue and spindle cell-rich non-adipose components, aiding in diagnostic differentiation [6]. This article highlights the diagnosis, management, and considerations of a rapidly growing SCL in the shoulder and neck region.

## Case presentation

**Figure 1:**
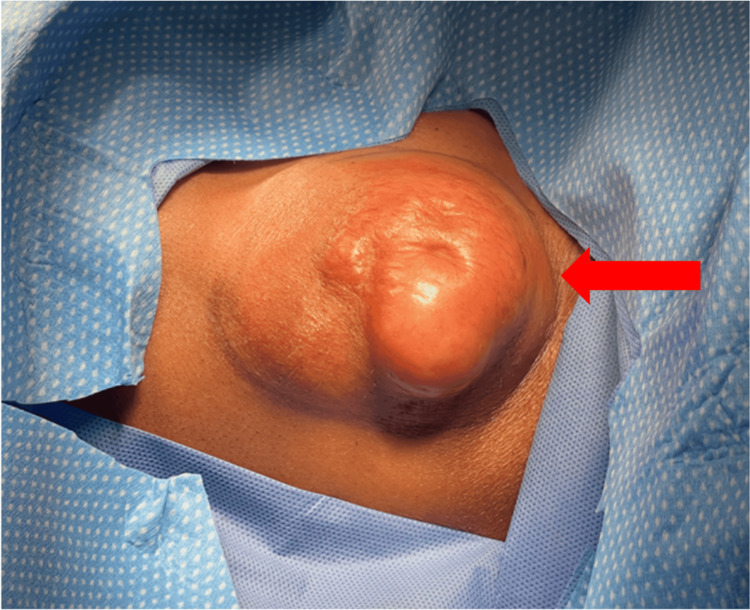
Appearance of the SCL mass located above the mid-to-distal clavicle prior to surgical incision Red arrow: SCL visualized on the patient’s skin. The top of the image is the patient’s cephalad direction. SCL: Spindle cell lipoma

In September 2024, a 64-year-old female patient presented with a palpable soft tissue mass overlying the left lateral clavicle, measuring approximately 4.0x4.0 cm during physical examination (Figure 1).

The patient reported noticing the mass one month prior to the initial visit but did not experience any associated systemic symptoms such as unintentional weight loss, fever, or chills. Her medical history was notable for well-controlled hypertension and hyperlipidemia, with a past surgical history of appendectomy and excision of a soft tissue cyst. She denied any history of smoking or recent travel.

**Figure 2:**
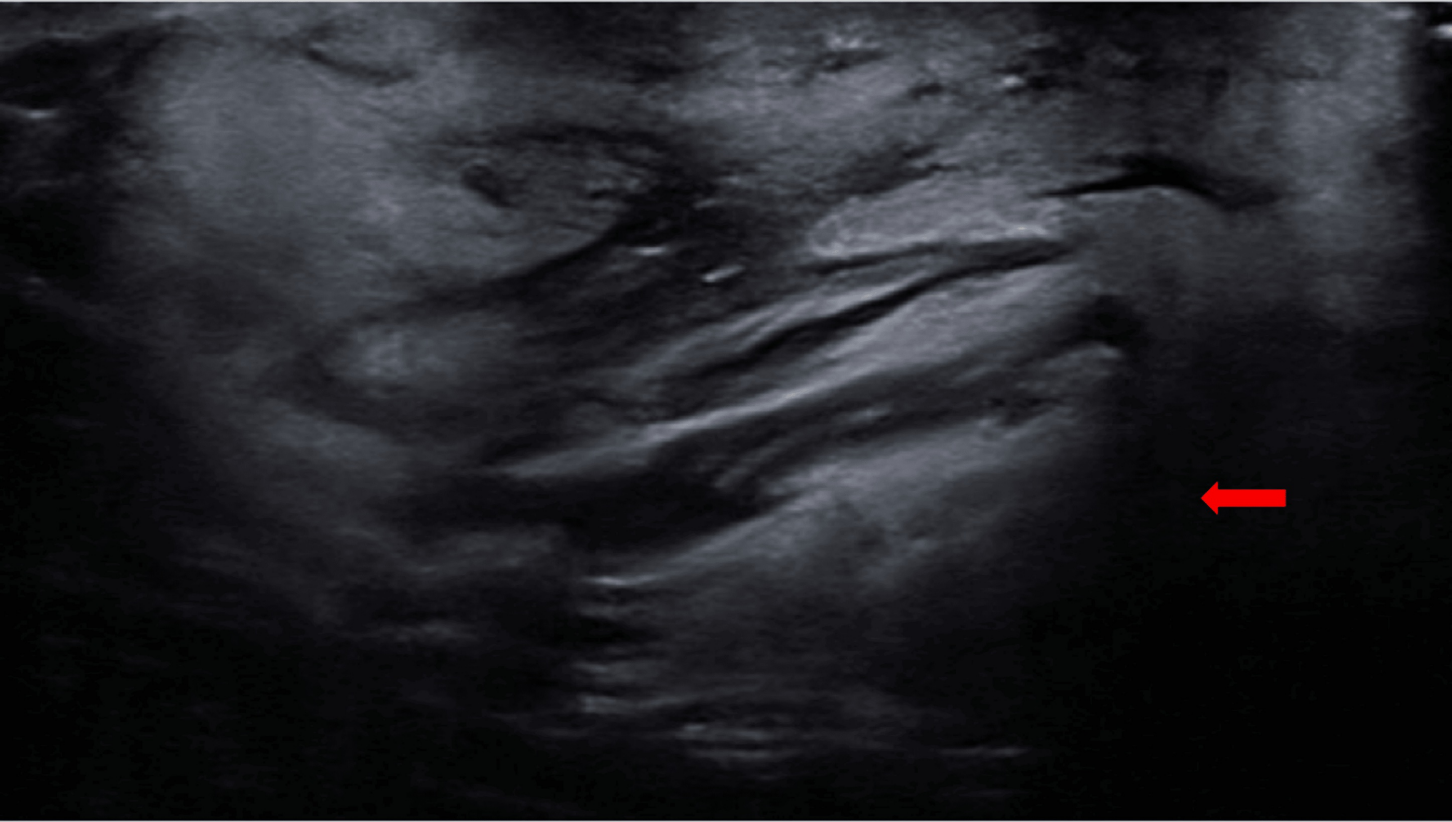
Initial ultrasound image of the left SCL mass Red arrow: SCL visualized via the ultrasound in a sagittal lateral view SCL: Spindle cell lipoma

Initial imaging with ultrasound revealed an ovoid mass above the mid-to-distal left clavicle, lateral to the left supraclavicular fossa, measuring 4.24x4.40 cm with a thickness of 2.20 cm (Figure 2). The mass appeared solid and heterogeneous internally with cords of isoechoic to hyperechoic tissue throughout.

**Figure 3:**
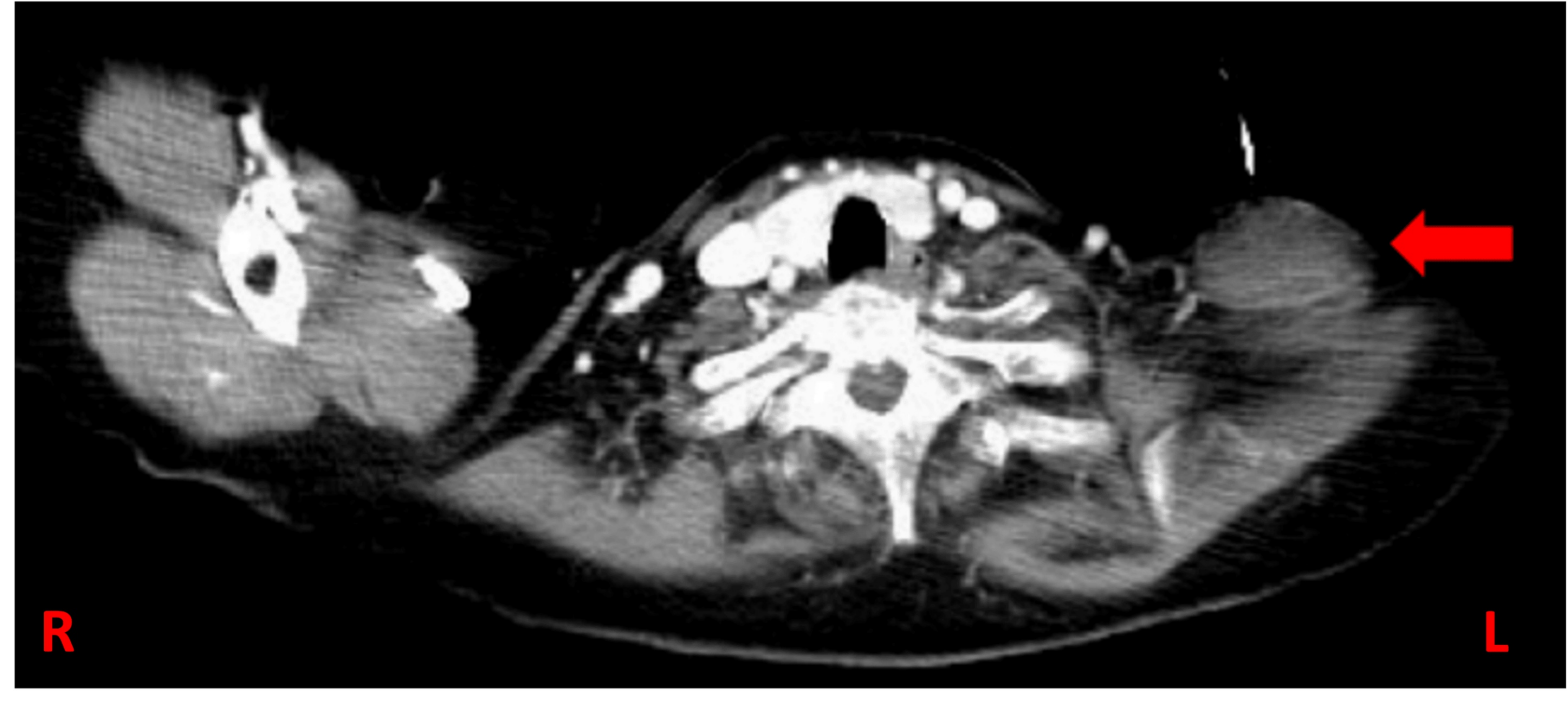
CT scan of the oval SCL lesion in the left supraclavicular region Red arrow: SCL visualized via CT scan SCL: Spindle cell lipoma; L: Patient’s left side; R: Patient’s right side

A subsequent CT scan identified a 3.9x2.3 cm oval lesion with poorly defined edges in the left supraclavicular region (Figure 3). The lesion appeared solid and heterogeneous, containing isoechoic to hyperechoic tissue strands. These findings suggested a neoplastic process, raising the possibility of both benign and malignant etiologies. 

**Figure 4:**
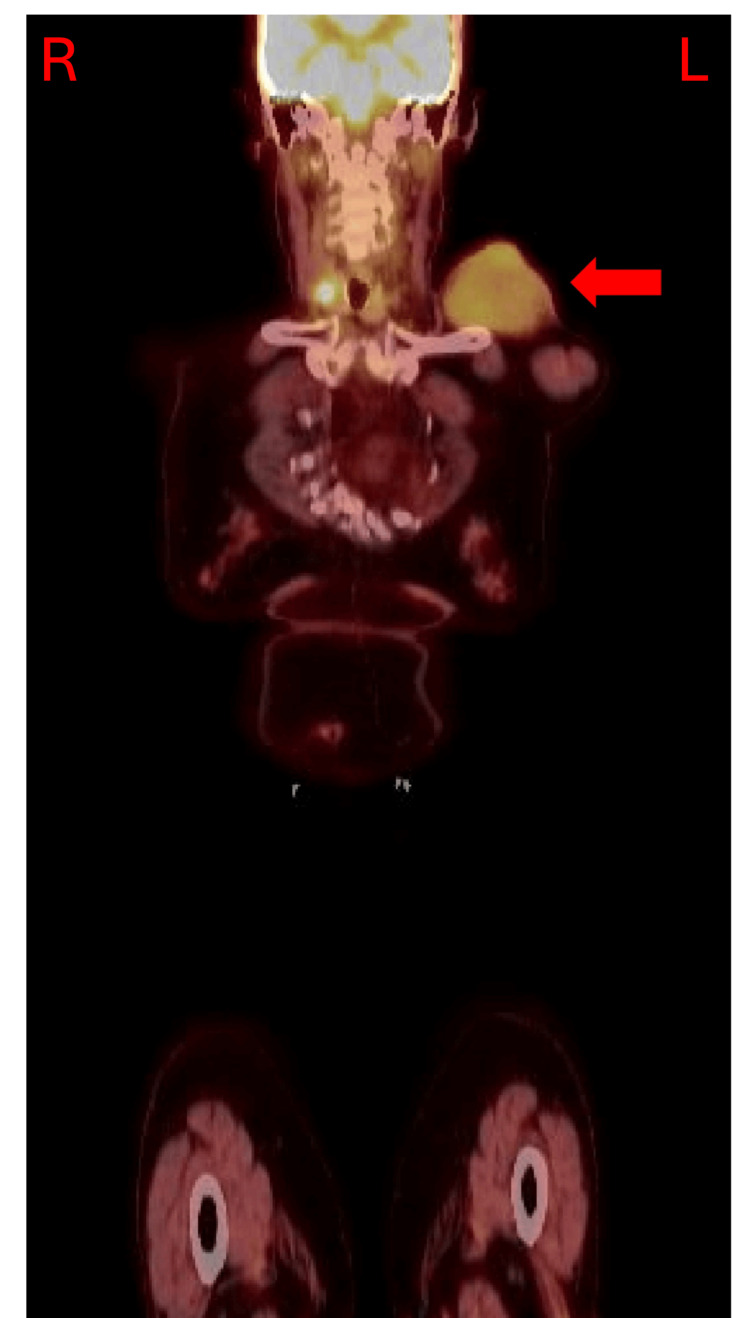
PET scan of the left supraclavicular mass Red arrow: SCL visualized via PET scan PET: Positron emission tomography; L: Patient’s left side; R: Patient’s right side

The patient did not follow up consistently due to significant hospitalization for unrelated health issues. When she returned for follow-up, she was referred to cardiothoracic surgery for further evaluation of the mass. Fine needle biopsy results indicated SCL with a benign appearance. Immunohistochemical analysis revealed the mass to be CD34 positive and vimentin positive. The analysis was negative for smooth muscle actin (SMA), desmin, and MUC4. Positron emission tomography (PET) showed a 5.6 cm left clavicular mass with mild avidity and standardized uptake value (SUV) of 3.7, suggestive of some malignant potential (Figure 4). 

Operative report

**Figure 5:**
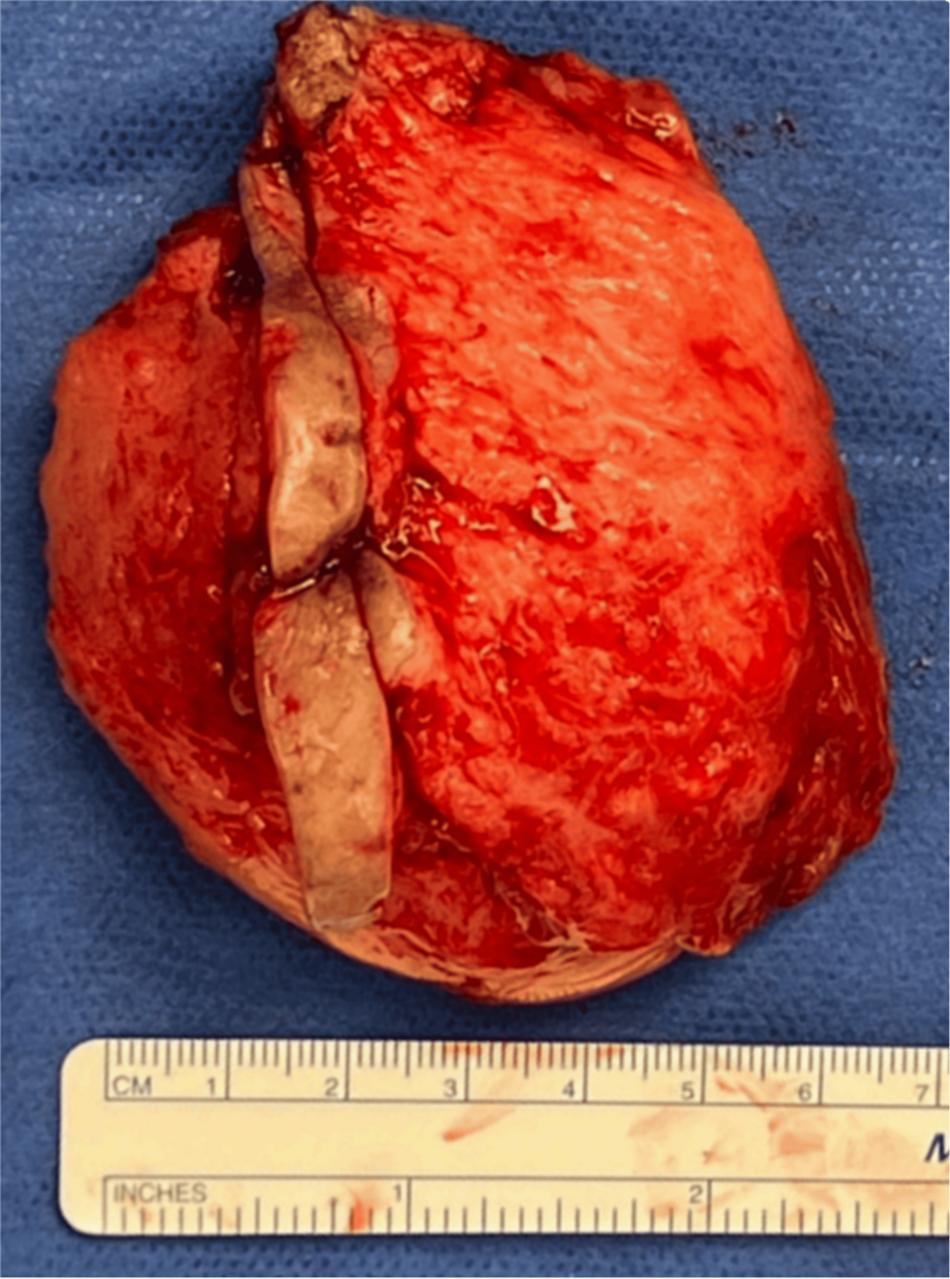
Gross appearance of the excised left supraclavicular bulk mass

Surgical excision of the mass was performed 10 months after the initial patient visit. An 11 cm incision was made over the left supraclavicular mass. The mass measured 7.0x6.0x5.0 cm and weighed 102 g (Figure 5). It was embedded in the deep muscle fascia as well as the superficial dermis, extending from the medial head of the clavicle medially to the lateral portion of the clavicle. The mass was large, firm, yellow in color, and adherent to the surrounding tissues, and was excised en bloc using both electrocautery and blunt dissection. The mass appeared well-encapsulated and was easily dissected from the surrounding structures. Perioperatively, there was no concern for muscular or neurovascular involvement. 

Pathology report

**Figure 6:**
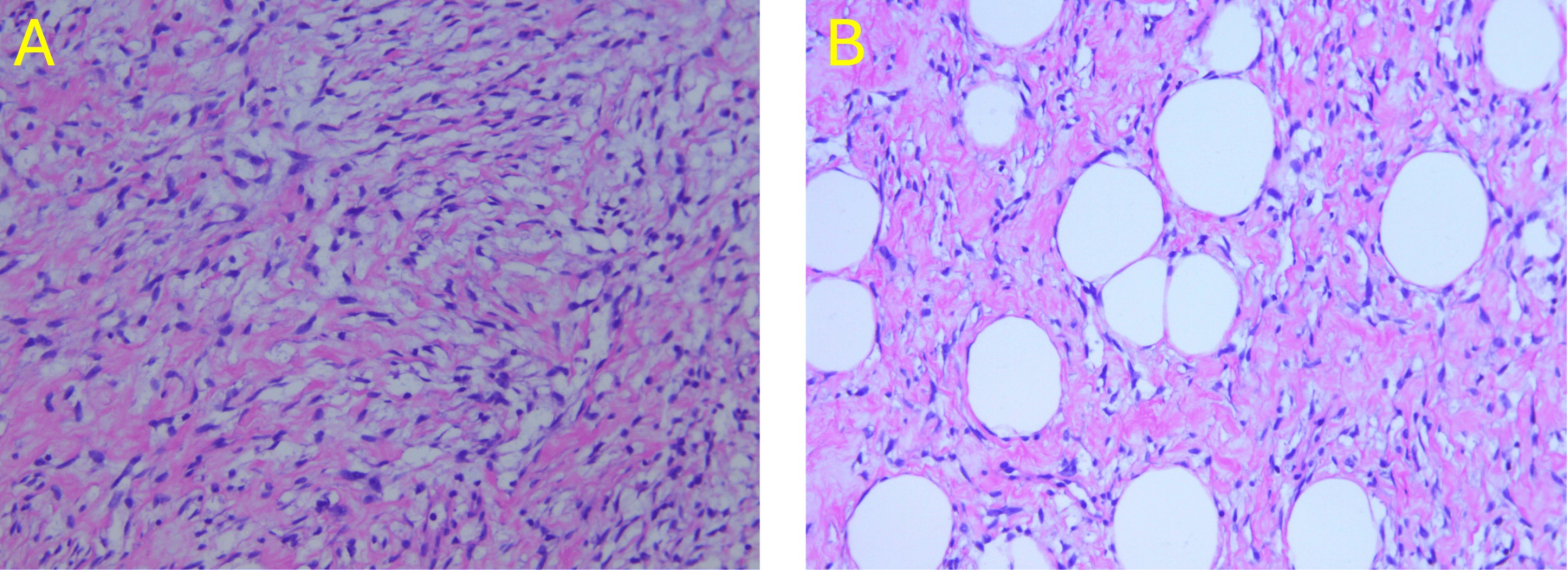
Frozen sections revealing a benign SCL without evidence of malignancy with the same immunohistochemical profile prior to surgical excision A: H&E stain showing spindle cells; B: H&E stain showing fat globules H&E: Hematoxylin and eosin; SCL: Spindle cell lipoma

Samples of the supraclavicular mass were examined pathologically both before and after surgical excision. Prior to surgical excision, the core needle biopsy exhibited the immunohistochemical profile as CD34 positive, vimentin positive, SMA negative, desmin negative, and MUC4 negative. Macroscopically, the mass exhibited a pinkish-gray myxoid appearance with a fibromyxoid stroma. These findings are consistent with a benign SCL. Following surgical excision, frozen sections from the suspicious areas of the mass, including the medial, posterior, superficial, deep, and inferior margins, were sent to pathology. The frozen sections revealed a benign SCL without evidence of malignancy with the same immunohistochemical profile prior to surgical excision (Figure 6). No gross invasion of the underlying structures, such as the sternocleidomastoid muscle, supraclavicular fat pad, or clavicle, were observed.

Post-operative follow-up

During the second-week follow-up, the patient was recovering without complications. The patient’s post-operative recovery was smooth, with no signs of hematoma, infection, or nerve damage. As of 10 months following the procedure, the patient has yet to return to the clinic with concerns of mass regrowth or new complications following surgery.

## Discussion

Our case presented an unusual manifestation of an already uncommon and rare subtype of lipoma. The patient initially presented with a small, soft mass over the clavicle. Imaging performed prior to the biopsy raised suspicion for a lipmatous mass but also suggested a possible neoplastic process due to the presence of poorly defined margins, raising concern for both benign and malignant etiologies. However, there was minimal concern for cardiothoracic or brachial plexus invasion, as neither imaging findings nor physical examination indicated involvement or associated symptoms. The tumor demonstrated rapid growth over the course of a year since its initial presentation. Both its location and progression are considered uncommon based on previous literature.

SCL is a rare subtype of lipoma, first described by Enzinger and Harvey in 1975 [2]. It typically occurs in men between the ages of 45 and 65 years old. In men, SCL is most often found in a "shawl" distribution, involving the shoulders and posterior neck. However, in women, SCL is frequently located outside this distribution and is under-reported. As recently as 2023, an updated review of SCLs found clinical features to be uncommonly present in skeletal and fascial tissue, as well as being slow-growing [7]. Previous case series hypothesize a more varied physical presentation in female patients, with a majority of cases occurring outside the shawl distribution. Additionally, SCLs were reported in slightly younger women, with a median diagnosis age of 51 years old [8]. SCL is characterized by a solitary, slow-growing, painless, and mobile tumor in the subcutaneous tissue. It is uncommon for SCL to infiltrate deeper structures such as fascia or muscles, and there are no reported cases of metastasis or malignant transformation. SCLs typically range in size from 1 cm to 4 cm, although larger tumors, up to 14-16 cm, have been identified [9].

Several subtypes of lipomas have been described: lipoblastomas (categorized by immature vs mature adipocytes), hibernomas (composed of brown vs white fat), angiolipomas (characterized by microvascular thrombosis), and pleomorphic lipomas (featuring "floret-like cells"). Differential diagnoses for giant lipomas include lipoblastomas and liposarcomas, with liposarcomas being the most common malignant variant. Distinguishing between lipomas and liposarcomas based solely on clinical appearance is impossible; therefore, surgical biopsy is mandatory to rule out malignancy. Regardless, lipomas tend to be less common in causing compression of the brachial plexus and local vasculature compared to neurofibromas and schwannomas in similar anatomical regions [10].

The pathophysiological origins of SCL remain unknown. Fine needle aspiration (FNA) cytology of SCL reveals that these tumors are composed of uniform spindle cells, fibroblasts, mature adipocytes, and collagen bundles [6]. Additionally, dendritic interstitial cells, mesenchymal cells, and mast cells are often intermixed. SCL cells are embedded in a myxoid matrix and stain positive for CD34, while staining negative for S100. Since CD34 is a marker for lipomas and lipoma-like sarcomas, further analysis of SCL typically shows negative staining for desmin, RB1, MDM2, and SMA. Molecular analysis indicates that SCL often exhibits deletions in chromosomes 13q and/or 16q. A key distinguishing feature of SCL is the rarity of lipoblasts within the tumor, which aids in differentiating it from other similar tumors [11].

On gross examination, SCLs tend to be oval-shaped, well-circumscribed masses, displaying a gray, white, and/or yellow color. This was consistent with the patient's debulked mass, being a 7.0x6.0x5.0 cm oval-shaped, yellow-colored tumor. As a subset of lipomas, SCLs exhibit varying amounts of fat content, depending on the ratio of fat (and adipocytes) to spindle cells. Most SCLs typically contain approximately 25-75% fat. The presence and identification of this fat within the tumor make radiographs, particularly MRI, the first-line imaging technique. However, some SCLs have a lower fat content, which impacts the fat-to-spindle cell ratio. These "low-fat" tumors are challenging to visualize with MRI because their lower fat components are isointense relative to skeletal muscle. Such masses can be mistaken for soft tissue tumors and/or more aggressive non-adipocytic sarcomas, including pleomorphic lipomas, fibrolipomas, collagenous fibromas, solitary fibrous tumors, sclerotic lipomas, liposarcomas, low-grade fibromyxoid sarcomas, and peripheral nerve sheath tumors like schwannomas and neurofibromas [12]. 

Alternatively, CT could be used to identify the well-defined borders and a mass with increased attenuation. Contrast can be employed to highlight the non-fat components that exhibit heterogeneous enhancement [1]. Recent studies report that SCLs typically exhibit consistent radiographic features, characterized by both adipose and non-adipose components within the lesion [13]. In contrast, atypical spindle cell masses or pleomorphic lipomatous tumors may appear radiographically normal or present as solid, heterogeneous, contrast-enhancing masses [14]. Due to the overlapping findings on imaging, a biopsy remains the definitive method for confirming and diagnosing SCLs. 

The optimal treatment for SCL is surgical excision [7,8]. Long-term prospective cohort studies (over 22 to 25 years) have demonstrated that local excision of SCL is curative, with recurrence being rare (1-2% after surgical treatment) [1,4]. Pre-operative FNA or post-operative histologic evaluation is recommended to confirm the diagnosis of SCL and to rule out other tumors, such as liposarcomas [4]. If infiltration had been present, as is sometimes seen with intermuscular lipomas, the patient might have experienced additional symptoms like peripheral neuropathy, necessitating more cautious resection [15].

In this particular case, the tumor's rapid growth, size, and supraclavicular location were initially concerning. Given its proximity to critical structures like the brachial plexus and subclavian artery, careful attention was necessary to protect them during any intervention. However, a preoperative FNA biopsy, followed by histological analysis, confirmed the tumor's spindle cell nature, which significantly reduced the likelihood of extensive adjacent structure involvement. This case is notable due to the patient’s sex, as SCLs are predominantly reported in male patients, typically occurring in the shoulders and posterior neck. Reports in female patients are minimal and often involve atypical anatomical locations. The presentation of an SCL in a female patient with a supraclavicular mass adds to the limited literature and highlights the importance of considering this diagnosis in atypical presentations, which can help guide appropriate management. Based on this case, we recommend that all atypical masses identified through imaging undergo FNA. If the biopsy confirms a diagnosis of SCL, the urgency of immediate intervention may be de-escalated. Nevertheless, in all confirmed cases of SCL, early and complete excision is advisable to limit further tumor growth and mitigate potential mass effect symptoms, such as pain or nerve compression, that could arise from the tumor pressing on surrounding tissues.

## Conclusions

This case highlights a rare presentation of an SCL in a 64-year-old female patient, characterized by its rapid growth and large size of 7.0x6.0x5.0 cm, located in the supraclavicular region. The unusual anatomical location and rapid progression prompted intraoperative pathological confirmation of the tumor subtype. This indeed showed characteristics consistent with SCL. Successful surgical excision was performed under general anesthesia. Histological examination of the mass revealed positive staining for CD34 and vimentin, and negative staining for SMA, desmin, and MUC4, which is consistent with previously reported findings for SCLs. This case report outlines the management of an SCL and offers insights for clinicians encountering shoulder and neck masses when considering a differential diagnosis. This case is unique because SCLs are rare in female patients and typically occur in the shoulders or neck of male patients. The supraclavicular location in a female patient adds to the limited literature and informs management of atypical presentations.
